# Serum progastrin-releasing peptide in pneumonia, chronic obstructive pulmonary disease and early-stage primary lung cancers

**DOI:** 10.11613/BM.2025.010702

**Published:** 2024-12-15

**Authors:** Gramos Begolli, Maja Lukić, Lada Rumora, Lorna Čorak, Andrea Vukić Dugac, Marko Jakopović, Miroslav Samaržija, Ilijan Tomaš, Jelena Knežević, Željko Debeljak

**Affiliations:** 1 Clinic of Medical Biochemistry, University Clinical Center of Kosovo, Pristina, Kosovo; 2 Clinical Institute of Laboratory Diagnostics, University Hospital Centre Osijek, Osijek, Croatia; 3 Faculty of Medicine, JJ Strossmayer University of Osijek, Osijek, Croatia; 4 Department of Medical Biochemistry and Hematology, Faculty of Pharmacy and Biochemistry, University of Zagreb, Zagreb, Croatia; 5 University Hospital Center Zagreb, Zagreb, Croatia; 6 School of Medicine, University of Zagreb, Zagreb, Croatia; 7 Oncology Department, University Hospital Centre Osijek, Osijek, Croatia; 8 Laboratory for Advanced Genomics, Ruđer Bošković Institute, Zagreb, Croatia; 9 Faculty for Dental Medicine and Health, JJ Strossmayer University of Osijek, Osijek, Croatia

**Keywords:** pro-gastrin-releasing peptide, early-stage lung cancer, inflammation, serum tumor markers

## Abstract

**Introduction:**

Higher concentrations of the small-cell lung cancer (SCLC) serum marker, pro-gastrin-releasing peptide (proGRP), in lung inflammations has been indicated in literature. The objective of this study was to compare serum proGRP concentration in pneumonia, chronic obstructive pulmonary disease (COPD) and early-stage primary lung cancers.

**Materials and methods:**

An observational study was performed to assess serum proGRP against other lung cancer markers in pneumonia, COPD and in stage 1/2 carcinomas. A total of 91 cases of pneumonia or chronic obstructive pulmonary disease (COPD), with 107 cases of early-stage lung adenocarcinoma (ADC), squamous cell carcinoma (SQCC) and 14 cases of neuroendocrine tumors (NET), including SCLC, were analyzed. Serum proGRP (Roche Diagnostics, Basel, Switzerland), cytokeratin 19 fragment 21-1, carcinoembryonic antigen, neuron-specific enolase and C-reactive protein were measured and compared. For the statistical analysis, Mann-Whitney U test, Kruskal-Wallis ANOVA, multiple linear and multinomial logistic regression modeling were used.

**Results:**

Compared to the early-stage ADC and SQCC, proGRP in pneumonia, COPD and in NET was higher (P ≤ 0.011 in all comparisons). In 11 cases of pneumonia and COPD, proGRP reached cut-off for SCLC of 100 ng/L. No clinically relevant differences between pneumonia or COPD and early-stage cancer were observed for other markers. Concentration of proGRP was associated with CRP (model coefficient was 0.20; P < 0.019) and both parameters contributed to classification of cases to pneumonia/COPD, ADC/SQCC, and NET categories (P < 0.004, in all cases).

**Conclusions:**

Concentrations of proGRP in pneumonia and COPD patients were higher than in patients in the ADC and SQCC early stages and could exceed the SCLC cut-off.

## Graphical abstract

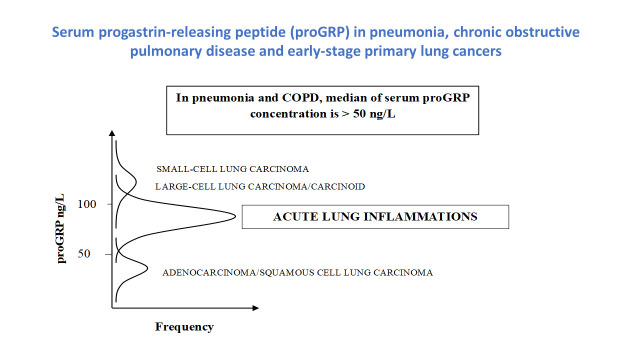

## Highlights

• In pneumonia and chronic obstructive pulmonary disease (COPD), median pro-gastrin-releasing peptide (proGRP) > 50 ng/L was measured

• Median proGRP < 40 ng/L was measured in the early-stage lung adenocarcinoma and squamous cell carcinoma

• Pneumonia and COPD patients may have higher proGRP cut-offs compared to small-cell lung cancer

• By using newly suggested rejection rules for the studied parameters, the analytical quality was not significantly improved

## Introduction

Lung cancer and lung inflammations like pneumonia or chronic obstructive pulmonary disease (COPD) may manifest themselves by similar initial symptoms, with inflammations often appearing concurrently with lung cancer (*1*). Unfortunately, inflammations may interfere in the lung cancer diagnostics by reducing diagnostic specificity of serum tumor markers (*2*, *3*). The most prominent example of the inflammation-related impact on tumor markers is carcinoembryonic antigen (CEA) which rise has been detected in pancreatitis, gastritis, cirrhosis, and hepatitis (*2*, *4*).

Small cell lung cancer (SCLC) and non-small cell lung cancer (NSCLC) are the major categories of lung cancer. The latter category is further subdivided in squamous cell carcinoma (SQCC), adenocarcinoma (ADC), large cell lung carcinoma (LCLC) and to carcinoid (*5*, *6*). The existing NSCLC markers do not have satisfactory diagnostic characteristics: CEA demonstrated a specificity of 68% and a sensitivity of 69%, whereas the cytokeratin 19 fragment 21-1 (CYFRA) exhibited a specificity of 89% and a sensitivity of 43% (*7*, *8*). However, most of the diagnostic performance studies involved different NSCLC stages: unlike stages 1 and 2, stages 3 and 4 of lung tumors do not pose a diagnostic challenge (*9*). Barouchos *et al.* assessed the concentrations of inflammatory indicators (C-reactive protein (CRP), erythrocyte sedimentation rate) and tumor markers (CEA, CYFRA, cancer antigen 125 (CA125), and carbohydrate antigen 19-9 (CA19-9)) in individuals with exacerbation of COPD (COPD-E) (*10*). It has been found that some tumor markers can exhibit increased concentrations in COPD-E. Besides that, only semi-quantitative data on lung inflammations-associated cancer markers’ alterations may be found (*11*).

Many studies proved the proGRP to be a useful marker in various aspects of SCLC diagnostics (*12*, *13*). The hormone gastrin-releasing peptide (GRP) is secreted by neuroendocrine cells, and it plays a role in the physiology of digestion and in the lung development (*14*-*16*). In SCLC, proGRP is secreted in an unprocessed form, and high concentrations of proGRP are found in the blood of patients with SCLC (*7*, *17*). Its diagnostic specificity and sensitivity for the detection of SCLC were assessed to be 95% and 84%, respectively (*8*). Furthermore, proGRP shows better clinical performance than neuron-specific enolase (NSE) in distinguishing between SCLC and NSCLC (*8*). Only rare subtypes of NSCLC like LCLC can produce proGRP: proGRP concentrations are higher exclusively in NSCLC tumors with neuroendocrine characteristics (*18*, *19*). Diagnostic properties of proGRP in SCLC are well-described but most of the diagnostic performance studies do not include pneumonia and COPD in the control group although some studies link higher proGRP to pneumonia, pneumonitis, COPD, tuberculosis and pulmonary fibrosis (*6*, *11*, *20*). Besides, higher proGRP can also be found in renal and hepatic diseases (*11*).

Gastrin-releasing peptide (GRP) itself appears to function as a potent proinflammatory mediator by inducing cell differentiation and/or activation of inflammatory cell precursors. Different immune response cells produce or react to GRP, which drives mast cell migration, degranulation, proliferation, and macrophage activation (*20*). It seems that proGRP, apart from being produced in lung inflammations, can induce lung inflammations. Although there is an indication of link between inflammation and serum tumor marker concentrations, impact of pneumonia and COPD on the serum tumor markers, in particular proGRP, issues additional research. This is reflected in the large differences in proGRP cut-off values used in differentiation of SCLC from the benign pulmonary diseases which vary from 50-100 ng/L between studies (*7*, *10*). The objective of this study was to compare serum proGRP concentration in pneumonia, COPD and early-stage primary lung cancers. Alterations of serum proGRP concentrations in the given states were compared to the alterations of CRP, CEA, CYFRA and NSE.

## Materials and methods

### Subjects

The observational study was conducted between 2020 and 2022 at the University Hospital Centre Zagreb, Croatia, and Osijek University Hospital Centre, Croatia, and it was open to male and female patients who signed informed consent and were referred to the hospital due to the suspected malignant lung disease. Patients were recruited based on physical and radiological (chest x-ray) examinations. Pneumonia was diagnosed according to the National Institute for Health and Care Excellence guidelines (*21*). The diagnosis of COPD patients was established according to the updated version of the Global Initiative for Chronic Obstructive Lung Disease practice guidelines (*22*). The establishment of a cancer diagnosis was made by a chest x-ray, computed tomography (CT) scan and lung needle biopsy in accordance with the World Health Organization classification of tumors (*5*). A total of 198 patients who met the inclusion criteria and signed the informed consent were enrolled in the study. The inclusion criteria were: age 18 years or older; pneumonia or COPD or lung cancer stage 1/2. Exclusion criteria were: cancer therapy; secondary tumors; stage 3/4 lung carcinomas, end stage kidney disease (glomerular filtration rate < 15 mL/min/1.73m^2^) and incomplete medical documentation. Due to a small number of cases, tumors of neuroendocrine origin were pooled to a single NET category. Due to technical difficulties, NSE was determined only in 149 cases. Table 1 provides an overview of the population under investigation. This study was conducted according to the Declaration of Helsinki, and it has received approvements by the corresponding Ethical committees (University Hospital Centre Osijek: R2-8262/2020; University Hospital Centre Zagreb: 8-1-17/50-2, No. 02/21 AG) (*23*).

**Table 1:** Demographic and clinical data

**Group**	**Pneumonia/COPD (N)** **(N = 91)**	**Early-stage lung cancer (N)** **(N = 107)**
Pneumonia	25	-
COPD-E	14	-
COPD-R	52	-
ADC	-	66
SQCC	-	27
NET (LCLC, SCLC, carcinoid)	-	14
Age (years, median (min-max))	70 (47-90)	63 (43-78)
Gender (male/female)	67/24	78/29
Smoking status* (smoker^†^/non-smoker)	61/28	73/34
*No data available for two participants. ^†^Ex-smokers included. COPD - chronic obstructive pulmonary disease. COPD-E - exacerbation of COPD. COPD-R - remission of COPD. ADC - adenocarcinoma. SQCC - squamous cell carcinoma. NET - neuroendocrine tumor. LCLC - large cell lung carcinoma. SCLC - small-cell lung cancer.		

Blood samples were collected from all participants to assess the concentrations of serum proGRP, CYFRA, CEA, NSE and CRP.

### Methods

Blood samples, taken during the routine diagnostic evaluation of suspected lung cancer, pneumonia or COPD, were drawn into plain collection tubes that did not contain any anticoagulant (Becton Dickinson, Plymouth, UK). Fasting was not mandatory. Blood samples were centrifuged at 1300xg for 10 min within 1 hour of collection. Serum samples were collected, aliquoted, frozen, and stored at - 70 °C until analysis. Concentrations of CEA, CYFRA, NSE and proGRP were determined using the electrochemiluminescence immunoassays implemented on the COBAS E601 analyzer (Roche Diagnostics, Basel, Switzerland). Concentration of CRP was determined by immunoturbidimetry on AU680 automatic chemistry analyzer (Beckman-Coulter, Brea, USA). Analytical assays were validated by the manufacturer and were regularly controlled using the internal and external quality assessment programs. Other laboratory test results like complete blood counts were not gathered.

### Statistical analysis

The R Statistical Software (ver. 4.2.0.; R Core Team 2021) was used for the statistical calculations (*24*). Kruskal-Wallis ANOVA has been chosen for the analysis of serum marker concentrations by clinical entities *i.e.* pneumonia, COPD, ADC, SQCC and NET. Due to the same reason Mann-Whitney U (MWU) test was used for assessment of the multiple pairwise comparisons of proGRP in the studied clinical entities. In this case, Benjamini-Hochberg false discovery rate (FDR) correction has been applied. Test of proportions was chosen for the analysis of nominal variables like smoker status and gender by clinical entities. Multiple linear regression (MLR) has been used for analysis of the association of proGRP with other serum markers and with age. In this case, Wald t-test has been used for determination of the regression coefficients’ significance. The significance of the complete MLR model was assessed by F test. Multinomial logistic regression (MNLR) was used for assessment of the association between studied markers and studied clinical entities: z-test has been used for determination of the regression coefficients’ significance. Significance of the complete MNLR model was assessed by classification accuracy determined using cross validation that was performed in the following way. Taking into account the small dataset size, 90% of the dataset has been used for the MNLR model training and 10% was left for the trained-model classification performance estimation and the described procedure has been repeated 20 times. P values < 0.05 were considered statistically significant.

## Results

**Figure 1:**
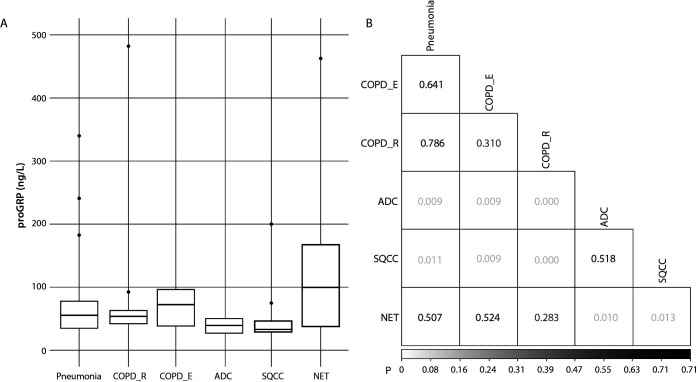
Box and whisker plots and the pairwise comparisons of pro-gastrin-releasing peptide (proGRP) of studied clinical entities (N = 198). A: proGRP in pneumonia, chronic obstructive pulmonary disease and different types of early lung cancer. For the sake of clarity y-axis has been truncated. B: false discovery rate corrected pairwise significances: legend contains the statistical significances presented in grayscale. ADC - adenocarcinoma. SQCC - squamous cell carcinoma. NET - neuroendocrine tumor. COPD-E - exacerbation of COPD. COPD-R - remission of COPD.

Figure 1 shows distribution of the serum proGRP values over different inflammatory diseases and different lung cancer subtypes. Differences in the serum proGRP concentrations between pneumonia, COPD and early NET were insignificant. This result, together with data presented in Table 2, confirms the anticipated association between increased proGRP and pneumonia or COPD. As expected, proGRP concentrations were higher (> 50 ng/L) in majority of NET cases, even in their early stages. But, more than a half of pneumonia and COPD cases were characterized by proGRP > 50 ng/L while 11 pneumonia and COPD cases had proGRP > 100 ng/L. At the same time, proGRP in only 2 of 4 SCLC patients and in 8 of 14 NET patients crossed the given cut-off of 50 ng/L, respectively (Table 2). On the other hand, differences between pneumonia and COPD, on one side, and ADC and SQCC, on the other side, were significant.

**Table 2:** Serum markers’ and demographic data distributions (N = 198)

**Parameter**	**Pneumonia** **(N = 25)**	**COPD-E** **(N = 14)**	**COPD-R** **(N = 52)**	**ADC** **(N = 66)**	**SQCC** **(N = 27)**	**NET** **(N =14)**	**P**
proGRP (ng/L)	55.7 (33.5-77.3)	72.0 (38.2-97.7)	53.3 (42.4-62.1)	39.1 (27.8-49.6)	32.4 (28.5-45.9)	98.6 (36.8-166.0)	< 0.001
CRP (mg/L)	143.4 (42.9-275.7)	66.3 (24.8-102.8)	3.9 (1.3-7.4)	2.10 (0.1-4.9)	5.30 (2.4-11.1)	3.3 (1.9-4.6)	< 0.001
NSE (µg/L) N = 149	4.7 (2.9-5.6)	3.8 (2.6-6.0)	10.2 (7.9-11.6)	8.7 (6.6-10.3)	9.4 (7.3-10.6)	8.0 (7.0-11.1)	< 0.001
CEA (µg/L)	2.6 (1.9-3.0)	3.8 (2.0-5.6)	3.4 (2.1-6.5)	3.3 (2.1-7.7)	2.6 (1.8-4.3)	2.5 (1.6-3.8)	0.093
CYFRA (µg/L)	3.2 (2.0-4.3)	2.4 (2.1-3.4)	1.9 (1.4-2.3)	2.3 (1.7-3.4)	2.1 (1.6-4.6)	2.4 (1.6-2.7)	0.004
Age (years, median (IQR))	70.0 (64.0-77.0)	72.0 (65.5-78.0)	63.0 (57.0-68.0)	64.5 (62.0-68.8)	64.0 (60.0-68.0)	59.0 (56.0-62.8)	< 0.001
Gender (male/female)	14/11	11/3	42/10	40/26	26/1	12/2	0.002
Smoking status* (smoker/non-smoker)	12/11	1/13	15/37	20/46	10/17	4/10	0.108
proGRP > 50 ng/L (N, ratio)	14 (0.56)	9 (0.64)	32 (0.62)	16 (0.24)	6 (0.22)	7 (0.50)	< 0.001
proGRP > 100 ng/L (N, ratio)	6 (0.24)	4 (0.29)	1 (0.02)	0 (0.00)	1 (0.04)	7 (0.50)	< 0.001
*Smoking status was not known in two cases. Quantitative results are presented as median (interquartile range). P < 0.05 was considered statistically significant. proGRP - progastrin-releasing peptide. CRP - C-reactive protein. NSE - neuron specific enolase. CEA - carcinoembryonic antigen. CYFRA - cytokeratin 19 fragment 21-1. COPD-E - exacerbation of COPD. COPD-R - remission of COPD. ADC - adenocarcinoma. SQCC - squamous cell carcinoma. NET - neuroendocrine tumor.							

It is worth noticing that CRP and proGRP concentrations were higher in COPD-E and pneumonia cases while in ADC and SQCC they were lower (Table 2). However, CRP was low in NET cases. This is more clearly depicted in Supplementary Materials (Figure 1) which provide insight in properties of proGRP and CEA in classification of the studied clinical entities. Majority of cases, no matter what class they belong to, group themselves near the graph origin. This graph indicates low accuracy of differentiation between pneumonia, COPD and early-stage lung cancer using these two markers: many pneumonia and COPD-E cases are characterized by proGRP values comparable to the values in early-stage NET. On the other hand, many COPD-R cases were characterized by CEA values comparable to the ones measured in majority of the early-stage ADC/SQCC cases.

Table 2 also presents distributions of other analyzed serum markers and demographic data: apart from CEA, all other parameters significantly differ among analyzed clinical entities. Differences in CYFRA and NSE detected between clinical entities, although statistically significant, were not clinically relevant since all values fell below the corresponding cut-offs. Differences in demographic parameters also deserve attention. Majority of COPD-E and pneumonia patients were older, and majority of NET patients were younger than the rest of the cohort: to determine the impact of age on associations between analyzed markers and studied clinical entities, age has been added to MNLR models. All studied clinical entities were dominated by males and smokers. The only exception represents the pneumonia category in which smokers and non-smokers were almost equally represented.

The developed MLR model of the proGRP was statistically significant (Table 3). The model indicates that, among studied numerical variables, only CRP was associated with proGRP. Contrary to expectations, proGRP was not associated to NSE, which is also considered to be a marker of NET. Also, MLR suggests that age does not make a significant impact on proGRP.

**Table 3:** Multiple linear regression of proGRP and multinomial logistic regression for differentiation of the early ADC/SQCC from the pneumonia/COPD or early NET (N = 149)

**Multiple linear regression (MLR)**			
	**Parameter**	**Coefficient**	**P**
	Intercept	43.21	0.415
	proGRP	-	-
	CRP	0.20	0.019
proGRP	NSE	- 1.88	0.221
	CEA	- 0.11	0.782
	CYFRA	- 3.82	0.315
	Age	0.65	0.397
**Multinomial logistic regression (MNLR)**			
	**Parameter**	**Coefficient**	**P**
	Intercept	- 2.47	0.161
	proGRP	0.04	0.001
	CRP	0.03	0.004
Pneumonia/COPD *vs*. ADC/SQCC	NSE	0.05	0.281
	CEA	- 0.05	0.260
	CYFRA	- 0.24	0.125
	Age	0.01	0.616
	**Parameter**	**Coefficient**	**P**
	Intercept	8.24	0.180
	proGRP	0.06	< 0.001
	CRP	- 0.04	0.421
NET *vs.* ADC/SQCC	NSE	- 0.13	0.613
	CEA	- 0.80	0.208
	CYFRA	0.29	0.626
	Age	- 0.20	0.032
MLR model’s P was 0.038 while the adjusted R2 was 0.046. MNLR model’s cross validation accuracy was 74.6%. P < 0.05 was considered statistically significant. proGRP - progastrin-releasing peptide. COPD - chronic obstructive pulmonary disease. ADC - adenocarcinoma. SQCC - squamous cell carcinoma. NET - neuroendocrine tumor. CRP - C-reactive protein. NSE - neuron specific enolase. CEA - carcinoembryonic antigen. CYFRA - cytokeratin 19 fragment 21-1.			

Multinomial logistic regression models are composed of two or more equations, depending on the number of entities aimed to be differentiated. For MNLR modeling, studied clinical entities were divided into three groups (NET, pneumonia/COPD, ADC/SQCC). In such settings, MNLR modeling produced two equations which coefficients and associated P values are given in Table 3. Accuracy of differentiation between groups was used for model performance assessment and is given in Table 3. proGRP and CRP were the variables that made difference between ADC/SQCC and pneumonia/COPD groups. On the other hand, proGRP and, to a smaller extent, age contributed to the model of NET *vs.* ADC/SQCC classification. Positive signs of proGRP coefficients and CRP coefficients from MNLR models increase the likelihood of pneumonia/COPD and NET over ADC/SQCC.

## Discussion

Presented results have shown that serum proGRP is higher in pneumonia and COPD. In concordance with that, proGRP was associated with the serum CRP. In these states proGRP may be even > 100 ng/L, *i.e.* as high as in SCLC, while in early-stage ADC and SQCC proGRP tends to be < 40 ng/L. On the other hand, other studied markers have not shown any clinically relevant differences between pneumonia, COPD and early-stage lung cancers.

As expected, Table 2 shows higher CRP in most patients with pneumonia and COPD. Besides the higher CRP, pneumonia and COPD were characterized by the higher serum proGRP. This is consistent with the suggested rise of proGRP in pneumonia and COPD. Moreover, proGRP and CRP were statistically associated (Table 3). The presence of circulating or locally produced pro-inflammatory cytokines, which cause the CRP elevation, may also be the cause of the serum proGRP rise. On the other hand, CRP and proGRP were lower in most cases of the early-stage ADC and SQCC. Hasan *et al.* have shown that CRP elevation in NSCLC is associated with tumor size and staging (*25*). This is in agreement with the results presented here taking into account that only early-stage lung cancers were studied. Serum CEA, CYFRA and NSE were below cut offs in pneumonia and COPD but also in early-stage lung cancers. Although Table 3 shows positive relationship between CRP and proGRP, this is less obvious among lung cancers (Tables 2 and 3). Differences in proGRP were not associated to differences in CRP in these cases. This finding might be explained by the fact that the tumor associated macrophages produce interleukins 4 and 10 that are anti-inflammatory cytokines (*26*). Maybe these cytokines locally block the production of proGRP. This relationship remains to be elucidated especially in light of some reports describing serum proGRP elevations in inflammations of other organs like brain and other lung diseases like fibrosis (*11*, *27*).

Figures 1A and 1B provide an insight into relationships between different types of cancers and inflammations on the grounds of proGRP. Pneumonia and COPD are tightly associated with NET while ADC and SQCC interrelated but differ from the COPD and pneumonia. Higher serum proGRP concentrations were found in NET but this result has been expected. How closely NET overlaps with pneumonia and COPD in terms of proGRP one may see in Supplementary Materials (Figure 1) and Table 2. In COPD-E proGRP may even reach values found in the early-stage SCLC and, in these cases, COPD-E may interfere with the SCLC diagnosis. NET were expected to be associated with increased serum NSE (*8*). Contrary to that, proGRP showed no association with the NSE (Table 3) what indicated different diagnostic information contained in these two NET markers. It is interesting to notice that NSE did not contribute to differentiation of early-stage ADC and SQCC cases from the early-stage NET cases (Table 3).

As a continuation of earlier studies showing higher proGRP serum concentration in benign non-inflammatory lung diseases and studies showing differentiation of NSCLC from the SCLC by proGRP, our study has shown the disparity in proGRP between the early-stage ADC or SQCC and COPD and pneumonia (*11*, *15*, *28*). While proGRP is established as a marker for SCLC even in its early stages, our findings suggest that proGRP may be seen as candidate tool to differentiate pneumonia and COPD from the most common NSCLC types in the early stage (*4*). As expected, CEA and CYFRA can not reliably differentiate early stages of most common types of NSCLC from pneumonia and COPD (Supplementary materials (Figure 1) and Table 3). The serum proGRP concentration was found to be a significant factor for distinguishing the early-stage ADC and SQCC from pneumonia and COPD (Table 3). According to results presented here proGRP concentration < 40 ng/L would indicate early-stage ADC/SQCC while serum proGRP concentration > 40 ng/L would indicate inflammation like COPD-E. In this case, CRP may help to discern pneumonia and COPD from NET. Besides proGRP and CRP, no other marker contributed significantly to classification of studied clinical entities.

Median proGRP concentrations were in the range of 30-40 and 50-70 ng/L in case of early-stage lung cancer, NET aside, and pneumonia or COPD, respectively (Table 2 and Figure 1A). It has been shown that these values vary between the cancer and inflammation subtypes. This result suggests that, depending on the test and control group composition, even the cut-off values used in diagnostic applications of proGRP may be different. Cut-off values of proGRP for SCLC vary between 50 and 100 ng/L (*7*, *10*, *29*). According to results presented in Table 2 if the control group recruited for study of the proGRP diagnostic performance contains pneumonia and COPD-E, the cut-off value for the differentiation of early NET, including SCLC, from acute lung inflammations should be closer to 100 ng/L. But even then, the diagnostic accuracy would be relatively low (Table 2).

Finally, some limitations of the study should be mentioned. Besides pneumonia and COPD other causes of elevated serum proGRP were not analyzed. Number of participants is relatively low. Although this sample size proved to be enough to show that the pneumonia and COPD-E compromise interpretation of serum proGRP results, the size is still too low to accurately assess diagnostic properties of proGRP in differentiation of major NSCLC subtypes from pneumonia and COPD. Although some differences in demographic parameters were detected they made no significant impact on presented results: some of these differences were clinically irrelevant while age differences didn’t make major impact on proGRP concentrations (Table 3).

## Conclusions

It has been shown that the proGRP concentration below 40 ng/L may be associated with the early-stage ADC or SQCC while the higher proGRP concentration, besides with NET, may be associated with acute inflammation like pneumonia or COPD-E. In COPD-E proGRP may even obtain values found in the early-stage SCLC. In contrast to other studied markers, the serum proGRP concentration significantly contributed to models for differentiation of early-stage ADC and SQCC and NET, pneumonia and COPD, but the relatively small sample size and study design limit the significance of this finding.

## Data Availability

The data generated and analyzed in the presented study are available from the corresponding author on request.
