# Corrigendum to: Serum progastrin-releasing peptide in pneumonia, chronic obstructive pulmonary disease and early-stage primary lung cancers

**DOI:** 10.11613/BM.2025.011201

**Published:** 2025-02-15

**Authors:** Gramos Begolli, Maja Lukić, Lada Rumora, Lorna Čorak, Andrea Vukić Dugac, Marko Jakopović, Miroslav Samaržija, Ilijan Tomaš, Jelena Knežević, Željko Debeljak

**Affiliations:** 1 Clinic of Medical Biochemistry, University Clinical Center of Kosovo, Pristina, Kosovo; 2 Clinical Institute of Laboratory Diagnostics, University Hospital Centre Osijek, Osijek, Croatia; 3 Faculty of Medicine, JJ Strossmayer University of Osijek, Osijek, Croatia; 4 Department of Medical Biochemistry and Hematology, Faculty of Pharmacy and Biochemistry, University of Zagreb, Zagreb, Croatia; 5 University Hospital Center Zagreb, Zagreb, Croatia; 6 School of Medicine, University of Zagreb, Zagreb, Croatia; 7 Oncology Department, University Hospital Centre Osijek, Osijek, Croatia; 8 Laboratory for Advanced Genomics, Ruđer Bošković Institute, Zagreb, Croatia; 9 Faculty for Dental Medicine and Health, JJ Strossmayer University of Osijek, Osijek, Croatia

## Abstract

• In pneumonia and chronic obstructive pulmonary disease (COPD), median pro-gastrin-releasing peptide (proGRP) > 50 ng/L was measured

• Median proGRP < 40 ng/L was measured in the early-stage lung adenocarcinoma and squamous cell carcinoma

• Pneumonia and COPD patients may have higher proGRP cut-offs compared to small-cell lung cancer

This is a correction of Biochem Med (Zagreb). 2025;35:010702. DOI: https://doi.org/10.11613/BM.2025.010702

Since the publication of the article, the authors have noticed an error in the Highlights section. The correct Highlights are presented above. We apologize to the authors for any inconvenience caused to the readers.

